# Structure–Property Evolution of Cubic and Gyroid PLA Scaffolds During In Vitro Degradation Under Physiologically Relevant Conditions

**DOI:** 10.3390/polym18161984

**Published:** 2026-08-14

**Authors:** Diana V. Portan, Lykourgos C. Kontaxis, Athanasia Tselepidi, George C. Papanicolaou, Leonard Azamfirei

**Affiliations:** 1 Laboratory of Biomechanics and Biomedical Engineering, Department of Mechanical Engineering and Aeronautics, University of Patras, 26504 Patras, Greece; portan@upatras.gr (D.V.P.); nasiatsel@gmail.com (A.T.); 2 Faculty of Medicine, George Emil Palade University of Medicine, Pharmacy, Science, and Technology of Targu Mures, 540142 Targu Mures, Romania; lykourgoskontaxis@gmail.com; 3 Department of Mechanical Engineering and Aeronautics, University of Patras, 26504 Patras, Greece; gpapan@upatras.gr

**Keywords:** 3D printed scaffolds, 3D biomaterials design, bone tissue regeneration

## Abstract

The design of innovative biomaterials increasingly aims to reproduce the structure, properties, and behavior of natural tissues. Biomimetic materials are generally considered to promote biointegration. In bone tissue regeneration materials, biomimicry and mechanical competence represent complementary design objectives whose relative importance depends on the clinical requirements and the expected timeline of bone regeneration. In the present investigation, two types of 3D-printed PLA scaffolds for bone tissue regeneration, featuring conventional cubic and biomimetic gyroid-based triply periodic minimal surface (TPMS) architectures, respectively, were evaluated. Their degradation behavior was investigated during immersion in a protein-rich cell culture medium under dynamic conditions at 37 °C, followed by mechanical characterization and analytical modeling of the property evolution. The cubic scaffolds exhibited approximately 210% higher apparent compressive modulus and more than fourfold higher apparent compressive strength compared with the gyroid scaffolds in the initial state. During 21 days of immersion, gyroid scaffolds showed progressive mass loss reaching approximately 13%, whereas cubic scaffolds exhibited a slight mass increase associated with fluid uptake. Both architectures experienced a reduction in mechanical properties; however, the gyroid structures showed faster structural deterioration due to their higher porosity, increased fluid accessibility, and greater fluid uptake and degradation-induced structural deterioration. The Residual Property Model accurately predicted the mechanical degradation of cubic scaffolds but was unable to predict the gyroid response due to the dominant contribution of mass loss and structural degradation. The overall results indicate a trade-off between the two architectural approaches, whereby enhanced biomimetic features are associated with reduced mechanical performance.

## 1. Introduction

Biomimetically engineered synthetic biomaterials are designed to reproduce selected characteristics of native tissues with the aim of improving their interaction with the biological environment [1]. The concept of biomimicry seeks to reduce the mismatch between synthetic materials and living tissues, thereby promoting a more favorable and long-lasting tissue–material interaction. The biomimetic character of a material may be expressed through different design features. These include chemical compatibility and bioactivity, structural and mechanical properties that resemble those of the target tissue, surface or internal architectures that support the transport of biological factors or therapeutic agents, and gradient characteristics or surface modifications that enable a smoother transition between the biomaterial and the surrounding tissue. The relative importance of these features depends on the intended application and the biological tissue to be replaced or regenerated [2].

The growing interest in biomimetic biomaterials has stimulated extensive research efforts toward the development of tissue-engineered scaffolds. Depending on the targeted application, biomimetic strategies may aim to replicate tissue gradients [3], extracellular matrix organization [4], vascular or lymphatic networks [5], and other microenvironmental characteristics that influence tissue development and regeneration. Attention has been given to the design of scaffolds with hierarchically organized architecture, as these more closely resemble the complexity of living tissues and may provide improved biological performance [6,7]. Likewise, fibrous scaffolds that mimic the organization of the extracellular matrix have been shown to influence cell attachment, proliferation, and differentiation, highlighting the important role of scaffold microarchitecture in regulating cellular behavior [8]. Beyond serving as structural support, biomimetic scaffolds may also recreate aspects of the cellular microenvironment that guide stem cell fate, as demonstrated by the use of microstructured extracellular matrix-inspired constructs capable of promoting tissue-specific differentiation and regeneration [9]. These developments reflect a broader trend in regenerative medicine toward the fabrication of constructs that not only replace missing tissue but also recreate aspects of the native biological environment in which tissue regeneration occurs. Consequently, the design of biomimetic biomaterials is inherently tissue-specific, requiring the selection and optimization of properties that are most relevant to the desired biological and functional outcome.

While soft-tissue substitutes may prioritize elasticity and deformability, and vascular implants place greater importance on surface characteristics that promote hemocompatibility and reduce thrombogenic responses [10], bone-related applications place particular emphasis on achieving adequate mechanical performance, as scaffolds must support physiological loads during tissue regeneration. However, higher porosity generally reduces mechanical strength, a factor that must be carefully considered [11,12].

Despite challenges related to their mechanical properties, biomimetic bone scaffolds can not only promote the osteogenic differentiation of stem cells by providing a bone-like microenvironment but also exhibit degradation rates that are closely matched to the ingrowth of new bone, thereby supporting effective tissue regeneration over time [13]. While numerous studies have focused on enhancing cell attachment, proliferation, differentiation, and seeding efficiency within bone scaffolds [14], the mechanical behavior of the synthetic structure has often been assessed using a relatively limited set of parameters. To balance biological functionality with structural integrity, few studies have investigated the influence of parameters such as porosity, relative density, and architectural organization on scaffold performance [15]. A comprehensive understanding of scaffold mechanics is essential, particularly for load-bearing applications where structural stability must be maintained throughout the bone regeneration process.

In hard tissue-associated applications, mechanical performance in correlation with microstructure is an appropriate approach. The emergence of biomimetic gradient scaffolds offers a promising solution to mimic the native interface’s complex gradient architecture by replicating the hierarchical organization and functional heterogeneity of the interface within a single construct, thereby achieving both structural biomimicry and functional tissue regeneration [16]. Material engineering strategies provide unique opportunities to investigate aspects of the structure–function relationship during new tissue formation in the laboratory and to predict the clinical outcome of specific medical treatments [17] as influenced by materials processing [18,19,20,21]. Building on the concept of correlating mechanical performance with scaffold microstructure, biomimetic multifunctional scaffolds have been developed to support osteochondral regeneration by integrating material design with functional tissue requirements. Such scaffolds reproduce the compositional, structural, and mechanical gradients of native osteochondral tissue, enabling simultaneous support of both bone and cartilage regeneration [22]. These developments highlight the importance of aligning scaffold architecture with the functional demands of the target tissue, illustrating how biomimetic design can bridge the gap between synthetic constructs and native tissue performance. Moreover, they demonstrate that biomimetic scaffold design must ultimately be evaluated according to its ability to preserve mechanical functionality while supporting tissue regeneration, thereby linking biological inspiration with structural performance.

A key requirement in biodegradable scaffold design is the ability to match degradation kinetics with the biological timeline of tissue regeneration [23]. In clinical bone repair, successful outcomes depend on maintaining mechanical support during early healing while allowing gradual load transfer to newly formed tissue. If degradation is too slow, it may hinder remodeling and tissue maturation, whereas excessively rapid resorption can compromise defect stability and healing success [24]. Consequently, scaffold degradation duration should be regarded as a clinically relevant design parameter that can be tuned to patient and defect-specific requirements.

In the present study, polylactic acid (PLA) was selected to manufacture the scaffolds due to its widespread use as a biocompatible and widely investigated thermoplastic polymer for biomedical additive manufacturing. Although PLA itself does not exhibit intrinsic osteoinductive or strong osteoconductive activity, its favorable processing characteristics, biodegradability, and compatibility with biological environments make it a widely investigated platform material for bone tissue engineering. In practical applications, PLA scaffolds are frequently combined with bioactive phases (such as calcium phosphate-based materials) [25], surface modifications, or biochemical cues [26] to enhance osteogenic responses. The biological compatibility of PLA has been extensively established, allowing its safe use in tissue engineering applications [27]. Nevertheless, before additional biofunctionalization strategies are introduced, understanding the intrinsic relationship between scaffold architecture, degradation behavior, and mechanical performance remains essential for the rational design of PLA-based bone regeneration systems.

The degradation of PLA-based scaffolds is generally initiated through hydrolytic cleavage of ester bonds within the polymer backbone. Water molecules diffuse into the amorphous regions of the polymer matrix, promoting chain scission and a progressive reduction in molecular weight. At the early stages of degradation, water uptake may act as a plasticizing mechanism by increasing polymer chain mobility and reducing stiffness, while simultaneously initiating hydrolytic degradation. With prolonged exposure, these processes may lead to loss of polymer integrity, surface erosion, fragmentation, and eventual mass reduction [28,29].

In porous architected scaffolds, degradation behavior is further governed by structural parameters such as porosity, pore interconnectivity, surface area, and fluid accessibility, which regulate water transport and the exposure of polymer surfaces to the surrounding environment [30]. Therefore, the degradation kinetics and mechanical evolution of PLA scaffolds cannot be considered independently of scaffold architecture.

Although FFF enables the fabrication of complex PLA scaffold architectures, the process may introduce fabrication-related limitations that influence the final structure–property response. Thermal degradation during repeated heating, residual moisture in the polymer filament, and anisotropy arising from the layer-by-layer deposition process can affect interlayer bonding and mechanical performance. Therefore, fabrication conditions and scaffold architecture should be considered together when evaluating the mechanical evolution and degradation behavior of 3D-printed PLA scaffolds [31].

Accordingly, the present study focuses on isolating the effect of scaffold architecture on mechanical evolution under physiologically relevant degradation conditions. The mechanical performance of pure PLA scaffolds has been previously studied under dry conditions [32] or in salt-based simulated body fluids [33]. The mechanical behavior of PLA-based composite scaffolds has also been recently reported both in the absence [34] and in the presence of protein-rich (α-MEM supplemented with 10% FBS) biological environments [35]. However, the architecture-dependent evolution of PLA scaffold structure and mechanical performance in protein-rich biological environments remains insufficiently explored and has rarely been systematically investigated, particularly under dynamic physiological-like conditions [36]. As previously emphasized by Portan et al. [37] in a comprehensive analysis of biomimetic testing environments, the assessment of biomaterials and biomedical devices should move beyond simplified salt-based simulated body fluids and instead employ physiologically relevant microenvironmental conditions that better replicate the complexity of body fluids in order to obtain more realistic degradation and performance responses under in vitro conditions. Therefore, the primary focus of this investigation is the mechanical performance of PLA scaffolds during immersion in cell culture medium, under dynamic rotation at 37 °C, with particular emphasis on how scaffold architecture influences structural integrity and load-bearing capacity. Besides the conventional scaffolds with square-shaped pores, gyroid biomimetic architectures have been manufactured for the present study, as they have recently emerged as advanced biomimetic scaffold designs for tissue engineering, offering a continuous, highly interconnected porous network that can be tuned to mimic the mechanical and transport properties of trabecular bone [38]. Moreover, semi-empirical analytical modeling provides a predictive framework for interpreting the evolution of scaffold mechanical behavior under environmental exposure, enabling reduction in trial-and-error experimentation and improved understanding of structure–property relationships during degradation-induced mechanical changes.

Finally, a clinical perspective is provided with the purpose of conferring a complex multidisciplinary approach to this study, making it useful in the design of a new generation of biomimetic biomedical materials.

## 2. Materials and Methods

### 2.1. Materials

Transparent PLA filament with a diameter of 2.85 ± 0.10 mm, named Ultimaker PLA, was purchased from Ultimaker (Geldermalsen, The Netherlands). The material had a density of 1.24 g∙cm^−3^, a melting temperature range between 145 °C and 160 °C, a tensile modulus of 2.34 GPa, and a tensile strength of 49.5 MPa, according to the manufacturer.

### 2.2. Scaffold Design and Fabrication

**Figure 1:**
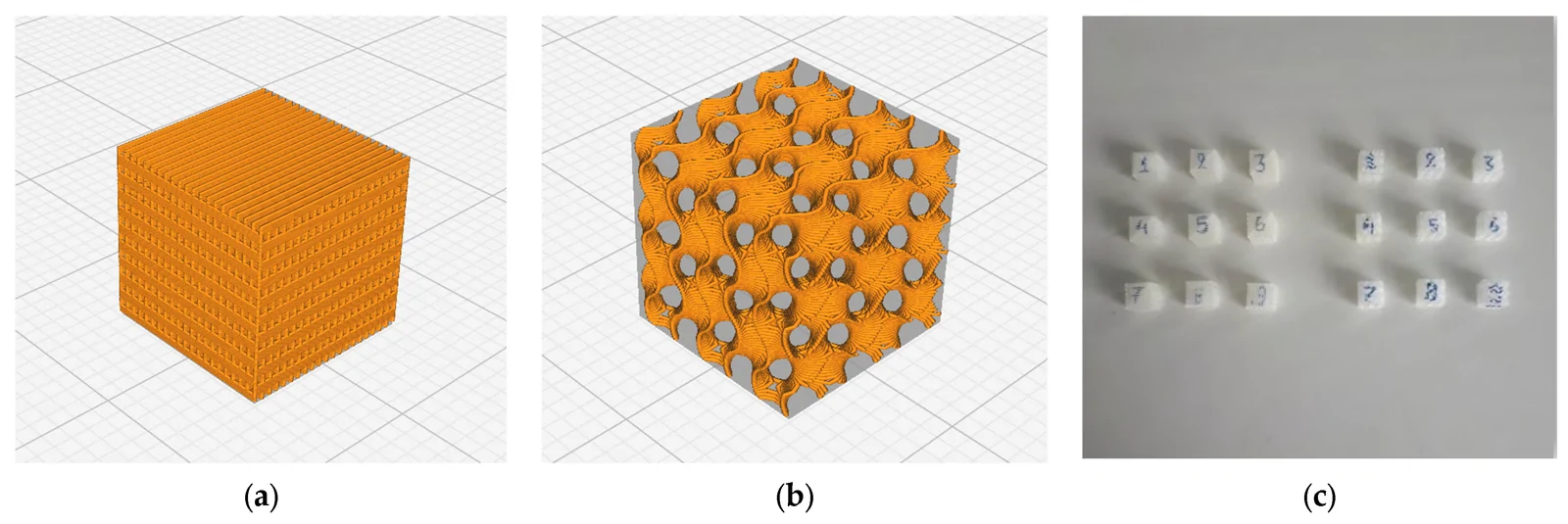
Scaffolds: schematic representation of (**a**) cubic scaffolds, (**b**) gyroid scaffolds, and (**c**) printed samples.

Scaffolds were manufactured using the fused filament fabrication (FFF) 3D printing system Ultimaker 2+ (Geldermalsen, The Netherlands). Digital models were prepared and sliced using Ultimaker Cura software v4.4.11. Two scaffold architectures were produced: (i) conventional cubic scaffolds with square-shaped pores, and (ii) gyroid-based triply periodic minimal surface (TPMS) scaffolds. Both types of scaffolds were fabricated with nominal dimensions of 10 × 10 × 10 mm^3^. All scaffolds were fabricated using a nozzle diameter of 0.25 mm, a bed temperature of 60 °C, and a printing speed of 30 mm/s. The first scaffold type featured a cubic architecture, assembled from cubic unit cells with strut dimensions of 0.22 mm and a pore size of approximately 500 µm, resulting in a theoretical porosity of 70%. The second scaffold type featured a gyroid architecture, utilizing the gyroid unit cell available as a built-in infill pattern in Ultimaker Cura 4.4.11. A line width of 0.22 mm and a line spacing of 1.5 mm were applied, yielding a theoretical porosity of 83% (Figure 1). The gyroid geometry is based on a triply periodic minimal surface (TPMS) structure, characterized by a continuous periodic surface dividing space into two interconnected regions. Unlike conventional lattice architectures composed of discrete struts and nodes, the gyroid design consists of continuously curved surfaces without sharp intersections, providing an interconnected three-dimensional pore network. In the present study, the gyroid geometry was generated using the corresponding infill pattern implemented in the slicing software, and the resulting architecture was fabricated under identical printing conditions as the cubic scaffolds to isolate the effect of topology on degradation and mechanical behavior.

The theoretical porosity of the cubic scaffolds was estimated from the infill density parameter set in Ultimaker Cura 4.4.11, according to: Porosity (%) = (1 − ρ_infill_) × 100, where ρ_infill_ is the volumetric infill density specified in the slicing software. An infill density of 30% was applied, yielding a theoretical porosity of 70%. Concerning the gyroid architecture, the characteristic pore aperture was governed by the line spacing parameter of 1.5 mm and the line width of 0.22 mm set in the slicing software, resulting in an effective characteristic pore dimension of approximately 1.28 mm. As the gyroid geometry is defined by a continuous, non-self-intersecting triply periodic minimal surface, discrete pore dimensions analogous to those of lattice architectures cannot be attributed; the aforementioned value therefore represents a linear approximation of the effective hydraulic pore aperture. Before the degradation experiments, the dimensions and mass of all fabricated scaffolds were measured individually to verify dimensional consistency and reproducibility. Scaffolds presenting dimensional deviations exceeding acceptable tolerances were excluded from the study. The initial mass values of all scaffolds used in the experiments are reported in Table 1.

### 2.3. Morphological Analysis (SEM)

In the present study, SEM analysis was employed primarily for qualitative assessment of architecture-dependent morphological changes and degradation mechanisms, while quantitative degradation evolution was evaluated through mass variation and mechanical characterization.

The surface morphology and structural changes were examined using scanning electron microscopy (SEM) (JEOL JSM-6610, JEOL Ltd., Akishima, Tokyo, Japan). Samples were sputter-coated with a thin gold layer prior to imaging. Observations were performed under high vacuum conditions at an accelerating voltage of 2 kV. Due to the layer-by-layer nature of FFF, different external faces of the scaffolds may exhibit distinct surface features associated with filament deposition and contact with the printing platform. Therefore, SEM observations were performed on representative scaffold regions selected to highlight degradation-related morphological changes.

### 2.4. In Vitro Degradation Environment

Scaffold degradation was investigated under dynamic immersion in α-MEM supplemented with 10% FBS at physiological temperature (37 °C). Samples were placed in a glass beaker containing 60 mL of Alpha-Minimal Essential Medium (a-MEM, BiochromKG, seromed, Berlin, Germany), supplemented with 10% fetal bovine serum (FBS, Biochrom), supplemented with 2.5 μg/mL Amphotericin B (Biochrom), 50 μg/mL Gentamycin (Biochrom), 1.5 mM β-glycerophosphate (Sigma, Athens, Greece), 50 μg/mL Ascorbic Acid (Sigma), and 50 μg/mL Penicillin–Streptomycin. This protein-rich medium was selected to provide a more physiologically relevant environment compared with simplified salt-based degradation solutions.

Prior to degradation experiments, all scaffolds were sterilized by ultraviolet (UV) irradiation. Each surface of the scaffold was exposed to UV light for 5 min, resulting in a total sterilization time of 20 min per specimen. All scaffolds were immersed simultaneously in the same glass beaker containing 60 mL of α-MEM-based cell culture medium. The degradation system was covered with aluminum foil to minimize evaporation of the culture medium during the 21-day immersion period while allowing limited gas exchange. Small openings were introduced in the foil to enable oxygen transfer and maintain an appropriate environment for the biological medium. Minimal volumes of fresh medium were added when necessary to compensate for evaporation losses and maintain the initial volume of 60 mL throughout the experiment. Therefore, the scaffolds remained exposed to the same degradation environment during the entire test period. The covered glass beaker was maintained under continuous agitation using a magnetic stirrer equipped with temperature control (magneto-thermo stirrer system). Two rotating magnetic bars were used to ensure uniform fluid motion, and scaffolds were subjected to continuous rotational flow throughout the experiment. The stirring speed was maintained at approximately 400–500 rpm, and the temperature was kept constant at 37 °C. The selected stirring speed was chosen to provide continuous medium movement and enhanced mass transport around the scaffold surface, promoting interaction between the polymer structure and the surrounding fluid. The stirring speed of approximately 500 rpm was selected to avoid stagnant conditions and to provide a controlled dynamic immersion environment, allowing investigation of architecture-dependent degradation behavior under increased fluid accessibility compared with static conditions. Although this setup does not directly reproduce the complex hydrodynamic conditions of the human body, dynamic fluid exposure is considered more representative than static immersion for evaluating scaffold–medium interactions. Previous studies have demonstrated that microvascular fluid velocities vary considerably depending on vessel diameter and physiological conditions, with reported values ranging approximately from 0.2 to 2.7 mm/s [39]. Therefore, the present dynamic condition was selected as an experimental approach to promote continuous fluid exchange and mass transport around the scaffold during degradation testing.

Scaffolds were immersed in the fluid for up to 21 days. Samples were retrieved at predetermined time points (0, 7, 14, and 21 days). At each time point, three specimens per architecture were removed for analysis. The three specimens of each architecture were considered experimental replicates and were immersed simultaneously within the same degradation medium to ensure identical exposure conditions. After removal, samples were gently rinsed to eliminate residual medium components and were surface-dried using a cold-air airflow source positioned approximately 10 cm from the scaffold surface. Drying was performed for 5 min on each side at room temperature, without external heating, to minimize any thermal influence on the PLA structure before mass and mechanical measurements. Oven drying was deliberately avoided to prevent any thermally induced structural or mechanical alteration of the PLA scaffolds prior to mechanical characterization, given the relatively low glass transition temperature of the material.

### 2.5. Mass Variation Analysis

The initial mass of each scaffold was measured individually before immersion and was subsequently used as the reference value for calculating its corresponding weight variation. The rinsing and drying procedure was applied to minimize the contribution of residual medium components and surface-adhered liquid to the measured mass variation. The obtained mass changes therefore reflect the variation in the scaffold mass after controlled removal of external moisture. Mass measurements were performed using a high-precision analytical balance. The percentage mass variation was calculated using:

(1) $$\mathrm{Weight}\;\mathrm{variation}\;\left(\%\right)=\frac{W_{t}-W_{0}}{W_{0}}\times 100$$

where *W*_0_ is the initial dry mass, and *W_t_* is the mass after immersion at time *t*. Measurements of the initial mass of the scaffolds are provided in Table 1.

### 2.6. Mechanical Characterization

Both cubic and gyroid scaffolds underwent compression tests, according to ISO 604, at a constant crosshead speed of 10 mm/min, using a Monsanto T20 uniaxial compression testing machine [Monsanto Company, St. Louis, MO, USA], at room temperature. Three specimens per scaffold type were tested at each interval (0, 7, 14, and 21 days) to assess the effect of degradation on the mechanical properties of the scaffolds. The scaffolds were placed between two parallel steel plates and compressed along their transverse axis. The scaffolds were compressed in the direction they were printed, and therefore no anisotropic properties were considered.

The apparent compressive modulus was estimated from the slope of the linear region of the stress–strain curve, and the apparent compressive strength was taken as the maximum stress recorded prior to the onset of the first plateau region. Nominal compressive stress and nominal compressive strain were calculated according to Equations (2) and (3), respectively:

(2) $$\sigma \;(\mathrm{MPa})=\frac{F\;(N)}{A\;({\mathrm{mm}}^{2})}$$

(3) $$\varepsilon =\frac{\Delta l}{l_{0}}$$

### 2.7. The Residual Property Model (RPM)

The Residual Property Model (RPM), developed by G. C. Papanicolaou et al. [40], is a predictive model used to estimate the time-dependent degradation of material properties resulting from damage. Its application is independent of both the material’s microstructure and the damage mechanism, making it suitable for a wide range of materials and degradation processes. The model has been successfully applied to several classes of materials subjected to various types of damage [41,42].

For damage induced by water absorption, the RPM relates the normalized residual property, defined as the ratio of the property at any time during the absorption process, P_r_(t), to the corresponding property of the dry material, *P*_0_, with the water uptake, *M(t)*, and to the ratio, s, of the property at the saturated state, *P_∞_*, to that of the dry material, P_0_. Thus, the evolution of the material property during moisture absorption can be predicted from the water absorption kinetics and the experimentally determined property values in the dry and fully saturated states [43,44,45,46].

The model is described by the following two relations:

(4) $$\frac{P_{r}}{P_{0}}=s+(1-s)\cdot e^{-sM}$$

(5) $$s=\frac{P_{\infty }}{P_{0}}$$

## 3. Results

### 3.1. SEM Images

**Figure 2:**
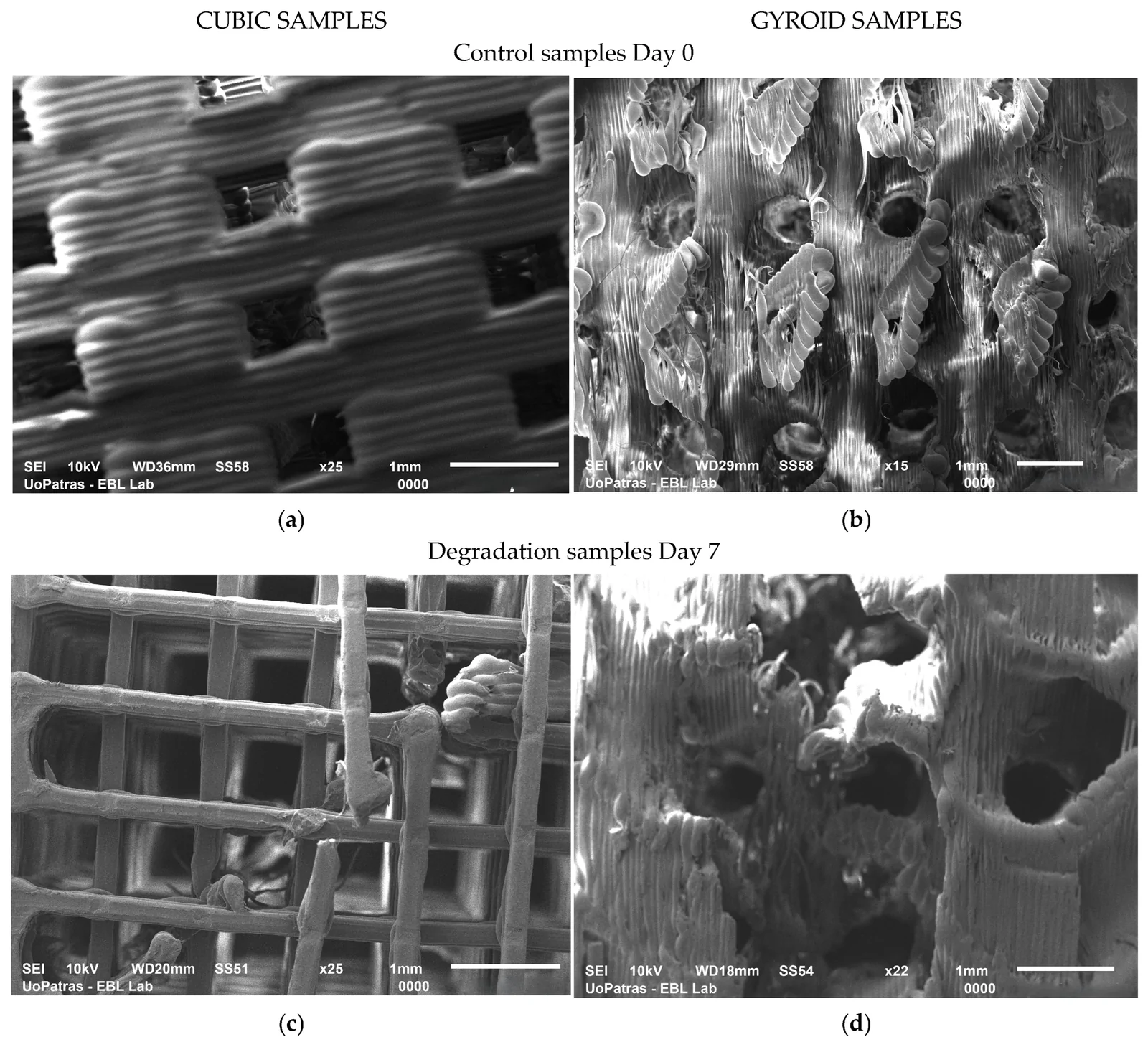

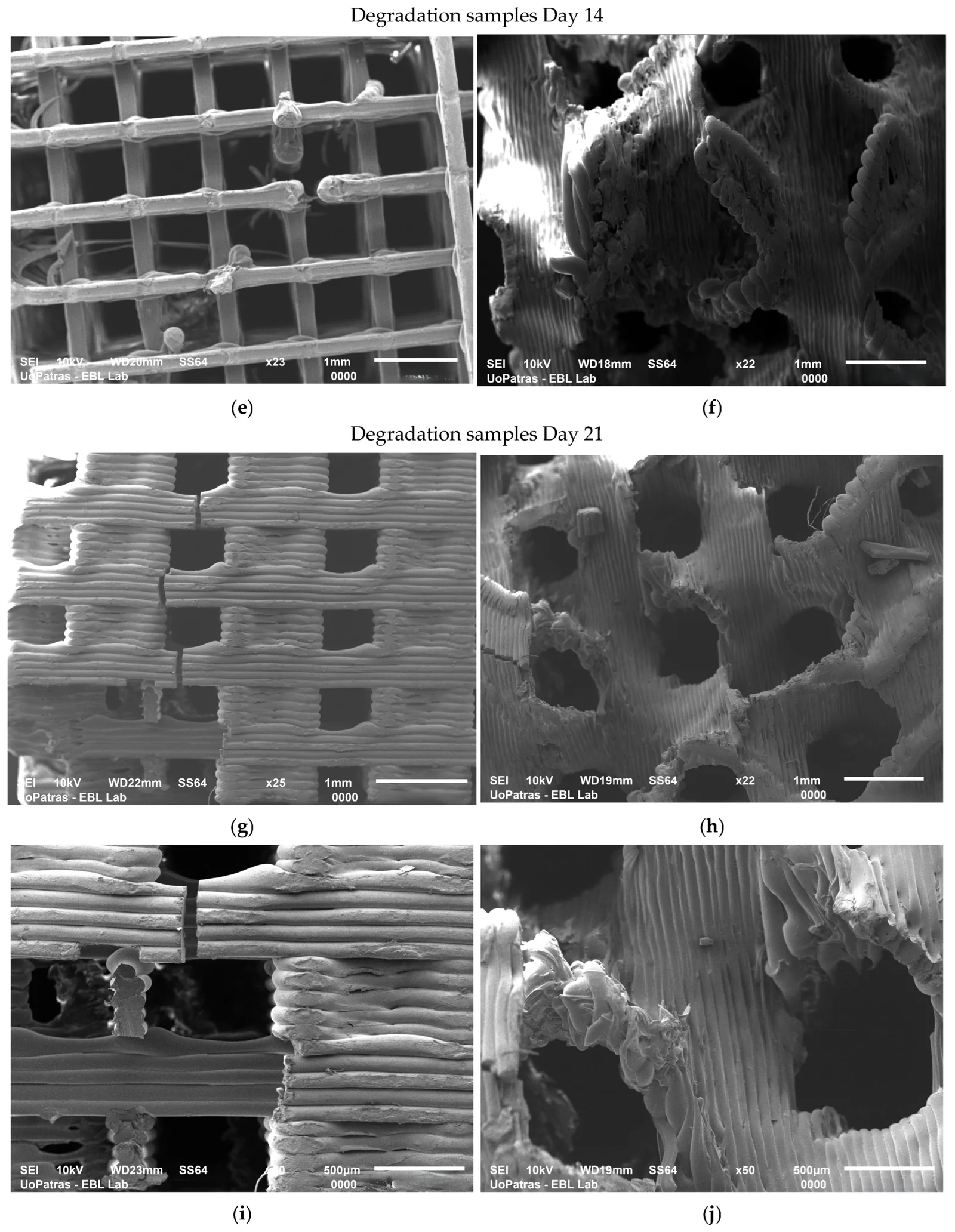
SEM images of the scaffolds before and after immersion up to 21 days (left column—cubic samples and right column—gyroid samples): (**a**,**b**)—no immersion; (**c**,**d**)—7 days immersion; (**e**,**f**)—14 days immersion and (**g**–**j**)—21 days immersion.

The SEM micrographs reveal pronounced microstructural changes in both scaffold architectures following immersion in the cell culture medium. The non-immersed control specimens (Figure 2a,b) exhibit well-defined and regular architectures characteristic of each design. The cubic scaffolds display a highly ordered lattice with clearly defined square-shaped pores and uniform struts. The deposited filaments show aligned printing traces, resulting in a compact and mechanically coherent structure. The gyroid scaffolds present a continuous triply periodic minimal surface (TPMS) architecture with interconnected and smoothly varying curvature. Compared to the cubic design, the gyroid structure exhibits a more complex and spatially continuous morphology, without distinct strut intersections. This results in a more heterogeneous microstructural appearance, although still highly regular in terms of periodicity.

After 7 days of immersion under dynamic rotational conditions at 37 °C, early signs of degradation are observed in both scaffold types (Figure 2c,d). In the cubic scaffolds (Figure 2c), localized filament discontinuities and initial crack formation are detected at strut junctions, indicating the onset of mechanical weakening. At this stage, no substantial surface plasticization is evident. In contrast, the gyroid scaffolds (Figure 2d) show more pronounced surface softening and localized deformation. The initial continuous filament deposition lines become partially disrupted, and localized flattening of material is observed, suggesting early-stage plastic deformation under combined hydrolytic and mechanical loading conditions.

After 14 days of immersion, degradation of the cubic scaffolds progresses primarily through crack propagation and progressive fracture of load-bearing struts (Figure 2e).

The gyroid scaffolds at 14 days exhibit attenuation of surface topographical features and evidence of localized material displacement along curved strut regions. The continuous architecture remains intact, but with reduced structural fidelity and increased surface irregularity, indicating progressive softening of the thermoplastic network. After 21 days of immersion, both scaffold types exhibit substantial structural degradation (Figure 2g–j). The cubic scaffolds (Figure 2g,i) retain partial architectural integrity but display extensive fracture accumulation and strut failure. In contrast, the gyroid scaffolds lose much of their characteristic TPMS features, with partial collapse of the interconnected network and a marked increase in open porosity. The original continuous morphology is no longer fully preserved, indicating advanced-stage degradation of the thermoplastic structure.

### 3.2. Degradation Rate

**Figure 3:**
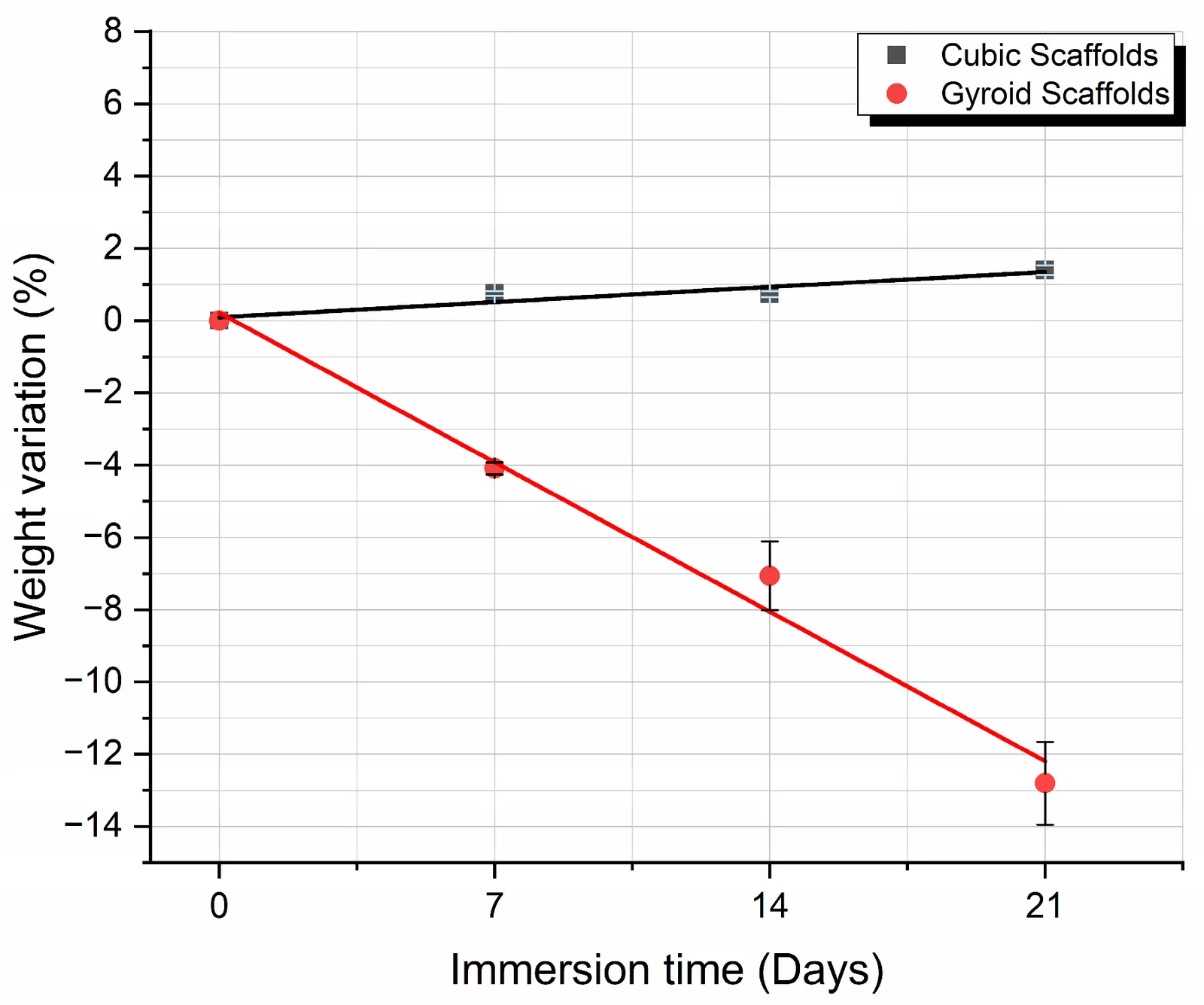
Weight variation in the two types of scaffolds: gyroid and cubic.

The percentage mass variation in the scaffolds as a function of immersion time is presented in Table 2 and Figure 3. The results indicate a time-dependent mass loss for the gyroid scaffolds, with a progressive decrease in mass throughout the immersion period. This trend is consistent with previously reported degradation behavior of TPMS-based scaffolds, where architecture and porosity have been shown to strongly influence fluid transport and degradation kinetics, leading to gradual and geometry-dependent mass changes under in vitro conditions [47]. In contrast, the cubic scaffolds exhibit a different response. During the first 14 days of immersion, they show negligible changes in mass, indicating limited overall material loss. After 21 days, a slight increase in measured mass is observed. Overall, no net mass loss is recorded for the cubic architecture over the experimental period; instead, a marginal mass gain is detected at the final time point. This behavior has been previously observed and will be developed in the Discussion section. At longer immersion times (2 weeks), the gyroid scaffolds exhibit a substantial mass loss compared to earlier time points, reflecting progressive degradation of the structure.

### 3.3. Mechanical Performance

**Figure 4:**
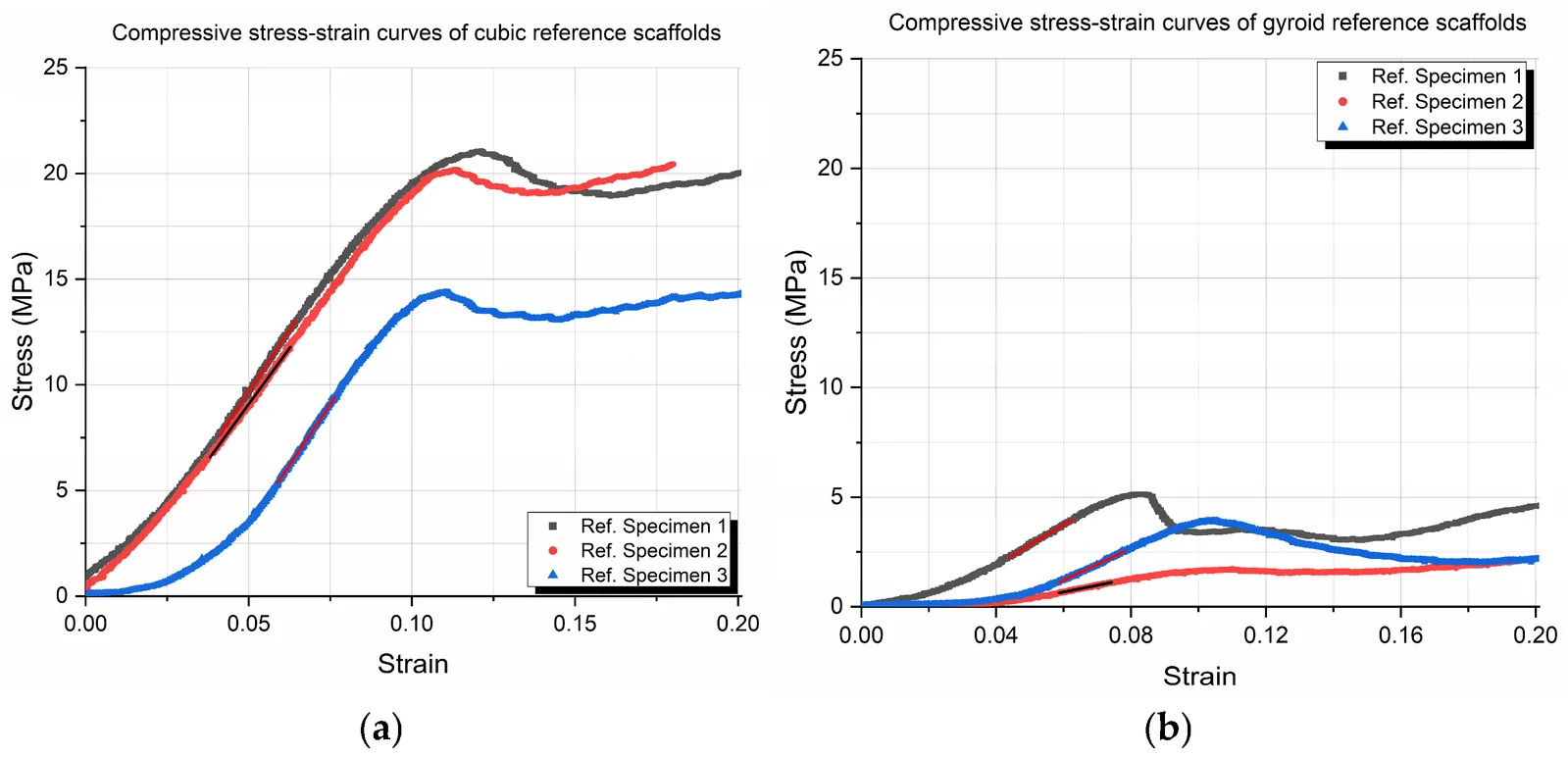
Stress–strain curves plotted after compression test for the control samples: (**a**) cubic scaffolds and (**b**) gyroid scaffolds.

Compressive stress–strain curves of the cubic and gyroid control scaffolds are plotted in Figure 4a,b. The graphs illustrate the deformation of the cubic and gyroid scaffolds in relation to the stress applied along their transverse axis.

The average apparent compression modulus and strength of the three measurements for the control samples are listed in Table 3. As observed, there is an extremely pronounced difference between the mechanical performance of the two types of scaffolds. The cubic scaffolds present an approximately 210% higher apparent compression modulus value compared to the gyroid scaffolds. Furthermore, the apparent compressive strength of the cubic scaffolds is more than four times higher than that of the gyroid samples. These values, in relation to the SEM images, confirm that an increase in the density of the architecture results in upgraded mechanical properties.

The mechanical properties obtained in this study should be considered in the context of porous bone regeneration scaffolds rather than dense bone tissue. Native cortical bone exhibits elastic moduli in the GPa range (approximately 16–22 GPa) [48], whereas cancellous bone presents considerably lower values, typically ranging from tens to hundreds of MPa depending on anatomical location and density [49]. Therefore, the apparent compressive moduli measured for the cubic (213.7 MPa) and gyroid (68.9 MPa) PLA scaffolds are comparable to the lower range reported for cancellous bone, while remaining substantially below cortical bone. This difference is inherent to highly porous scaffolds, where reduced relative density is required to facilitate cell migration, nutrient transport, and tissue ingrowth. Similar architecture-dependent trends have been reported for TPMS-based scaffolds [50], where increasing porosity and interconnectivity improve biomimetic characteristics but reduce initial mechanical performance.

**Figure 5:**
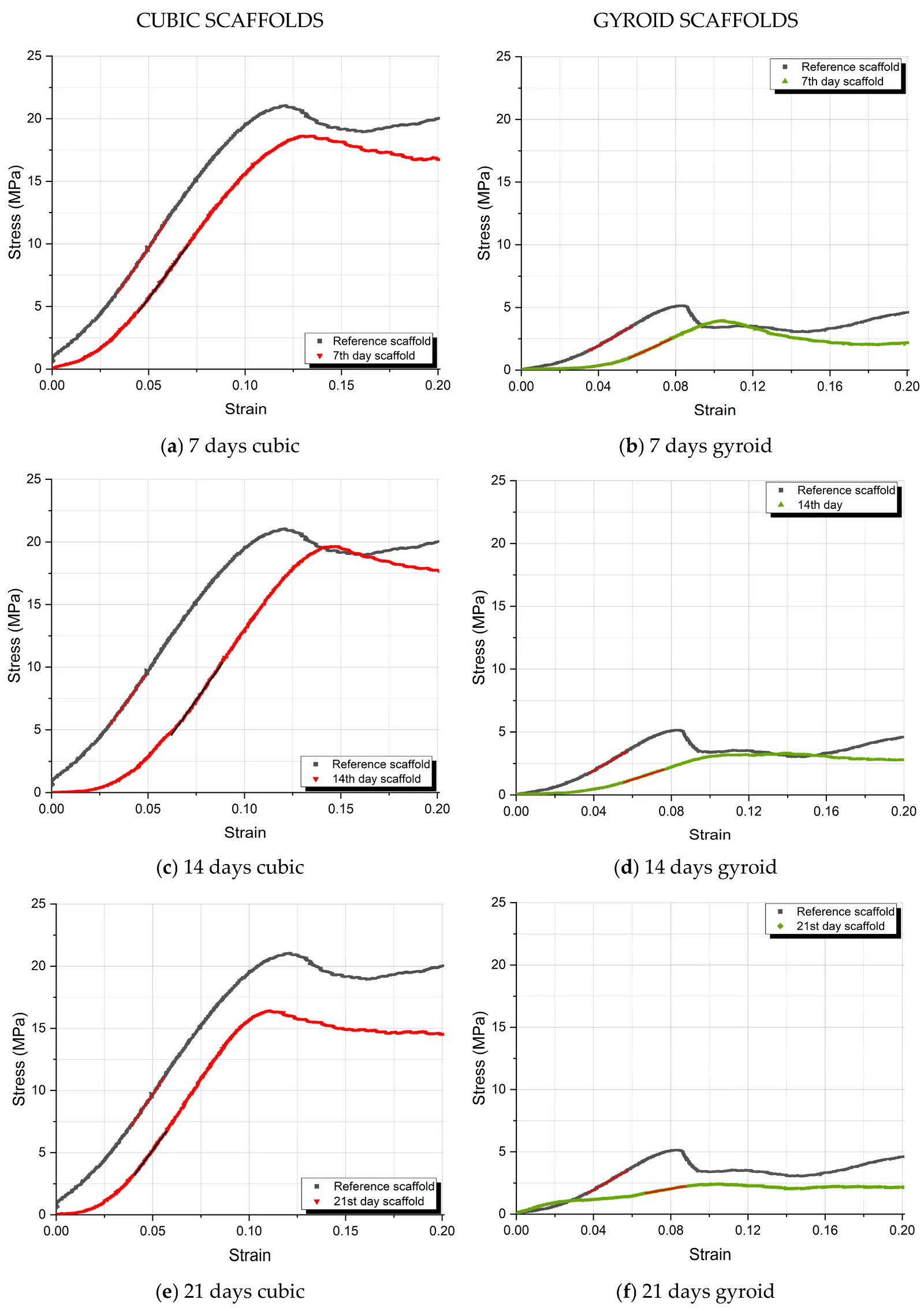
Comparative stress–strain curves of reference and (**a**) 7th-day cubic scaffold, (**b**) 7th-day gyroid scaffold, (**c**) 14th-day cubic scaffold, (**d**) 14th-day gyroid scaffold, (**e**) 21st-day cubic scaffold, (**f**) 21st-day gyroid scaffold.

Representative compressive stress–strain curves of cubic and gyroid scaffolds on days 0, 7, 14, and 21 are shown below (Figure 5), illustrating their mechanical response under uniaxial compression. The initial region of the curves, extending to a nominal strain of approximately 0.025, exhibits a low-stiffness response, with relatively small, applied loads resulting in comparatively large deformations. This behavior is more pronounced in the immersed specimens and can be attributed to moisture uptake, which reduces the effective stiffness of the polymer network, as discussed in detail below.

The compressive stress corresponding to failure is identified at the maximum stress value preceding the onset of a plateau region, where increasing deformation occurs with little or no additional increase in load-bearing capacity. This post-peak behavior is consistent with the collapse of porous structures, as reported in studies on trabecular bone mechanics, where initial strut failure leads to progressive densification and a transition toward a more compact, load-bearing configuration [51].

Failure in the cubic scaffolds occurs at strains of approximately 10–15% of their initial height, whereas the gyroid scaffolds fail at lower strains, typically between 8 and 12 results indicate that the gyroid architecture, characterized by higher porosity and a more open interconnected network, reaches structural failure earlier under compressive loading. This is attributed to the reduced capacity of the gyroid structure to redistribute and sustain applied loads, leading to earlier loss of mechanical integrity.

**Figure 6:**
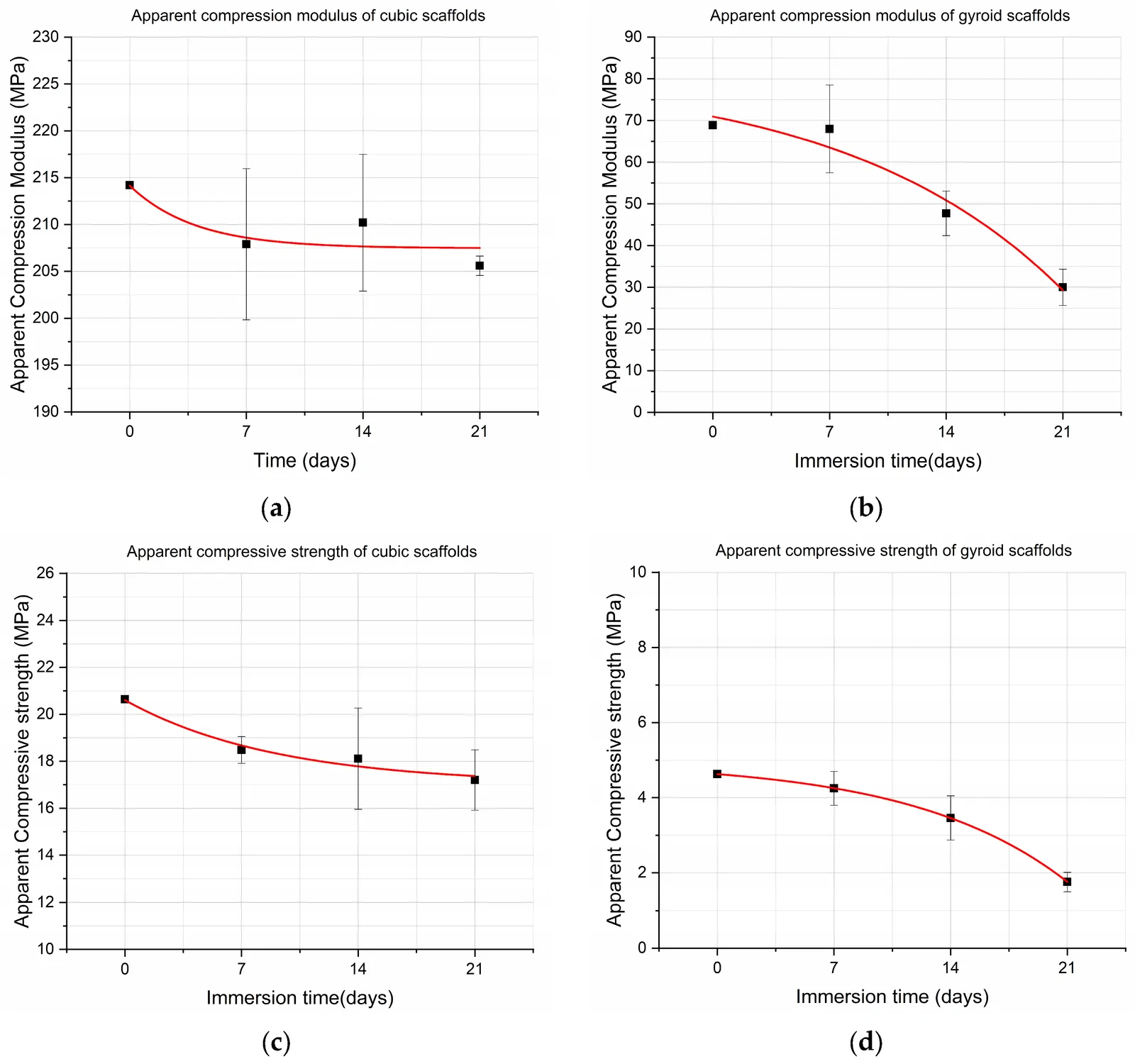
Apparent compression modulus as a function of immersion time for (**a**) cubic scaffolds and (**b**) gyroid scaffolds, and apparent compressive strength as a function of immersion time for (**c**) cubic scaffolds and (**d**) gyroid scaffolds.

The values of the apparent compression modulus of elasticity and the apparent compressive strength of the two scaffold structures over the course of 21 days in cell culture medium may be seen in Figure 6. As observed, both the apparent compression modulus and apparent compressive strength exhibit a decreasing trend with increasing immersion time. This reduction appears to follow a continuous decay, although no abrupt fluctuations are observed throughout the degradation period. The corresponding results illustrate the influence of the aqueous, dynamically stirred environment on the mechanical response of both scaffold architectures.

For the cubic scaffolds, both the apparent compression modulus and apparent compressive strength exponentially decrease with immersion time. The lowest modulus is recorded on day 21, whereas the highest value corresponds to day 0, prior to immersion in the degradation medium. Since the modulus is a measure of material stiffness, its reduction indicates a progressive loss of structural rigidity. Consequently, for a given applied stress, the resulting strain increases, reflecting a more compliant mechanical response of the scaffold over time.

In contrast, the apparent compression modulus and apparent compressive strength evolution of the gyroid scaffolds do not follow the same monotonic trend as observed for cubic architecture. Instead, both properties exhibit a parabolic decrease with immersion time. This behavior can be attributed to the significantly higher surface area exposed to the liquid in the gyroid structure, which enhances fluid interaction and accelerates degradation-related phenomena. As a result, more pronounced deterioration of mechanical properties occurs during immersion.

### 3.4. Residual Property Model Results

**Figure 7:**
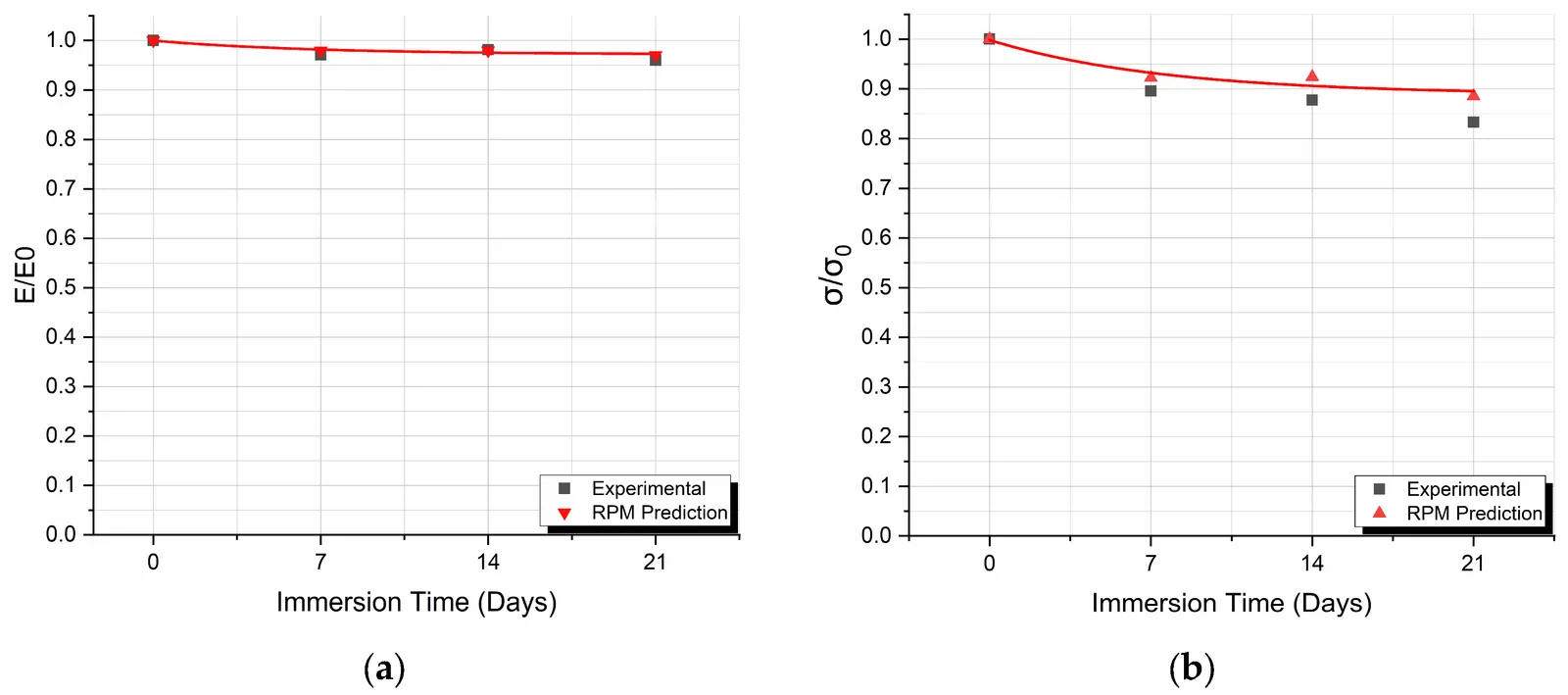
Predictions of the RPM for the reduced properties of: (**a**) Apparent compression modulus and (**b**) Apparent compressive strength.

The application of the model requires knowledge of the time-dependent water absorption, *M(t)*, of the material under investigation, together with the experimentally determined values of the property in the dry state, *P*_0_, and in the fully water-saturated state, *P_∞_*. The application of the RPM in the case of cubic scaffolds may be seen in Figure 7a,b. 

Therefore, within the investigated conditions, the RPM should be considered applicable for scaffold architectures where moisture uptake is the dominant mechanism affecting mechanical properties, whereas architectures undergoing significant mass loss and topology-driven structural degradation require additional degradation descriptors beyond water absorption kinetics. In the case of gyroid scaffolds, the RPM fails to predict the residual properties, and this can be attributed to the fact that the model considers moisture absorption as the main mechanism controlling property evolution, whereas gyroid scaffolds undergo additional architecture-dependent degradation phenomena involving mass loss and structural deterioration.

In PLA-based porous scaffolds, hydrolytic degradation is initiated by water diffusion and ester bond cleavage, resulting in polymer chain scission and progressive deterioration of structural integrity. However, in architected systems fabricated by FFF, the rate and consequences of these molecular processes are strongly influenced by scaffold topology, which governs fluid accessibility and damage propagation. Since degradation is strongly influenced by the internal geometry, porosity alone is insufficient to predict the degradation kinetics. Instead, the pore topology, the tortuosity, and the connectivity govern the fluid transport and the hydrolytic accessibility. Among the architected materials, the cubic lattice structures and the gyroid (TPMS) structures represent two fundamentally different transport regimes, since the cubic lattices exhibit more restricted diffusion pathways, while the gyroid structures provide fully interconnected 3D transport networks.

As a consequence of the above scaffold characteristics, it was experimentally observed that the cubic scaffolds exhibited negligible mass loss, limited structural change, and stable integrity over time, while, on the contrary, the gyroid scaffolds showed significant mass loss, visible structural degradation, and progressive fragmentation. This explains why the RPM fails in the case of gyroid scaffolds, since the model takes into account only the moisture absorption of the material, whereas the residual property of the gyroid scaffolds is governed by advanced degradation and significant mass loss, a mechanism unrelated to moisture absorption. This finding has major implications, since scaffold degradation can be engineered via the topology; the gyroid structures enable controlled biodegradation, and the cubic structures may be utilized for long-term stability applications. In Table 4, the predicted RPM values versus the experimental ones are presented, along with the percent difference between them. It is evident from the percent difference values that the RPM predicts the degradation of the cubic scaffolds extremely well, with a maximum deviation of 5.9%.

## 4. Discussion

### 4.1. Structure Correlation to Function

The experimental results demonstrate that scaffold architecture is the primary factor governing the degradation-induced evolution of mechanical performance under the investigated conditions. The observed differences between cubic and gyroid scaffolds are consistent with previous studies reporting that scaffold geometry strongly influences both the initial mechanical response and the subsequent degradation behavior of FFF-fabricated porous structures [52,53,54]. In this context, the present work is a case study analyzing the mechanical behavior of two types of 3D-printed PLA scaffolds, cubic and gyroid, respectively, when subjected to a relevant environment (immersion in SBF-enriched cell culture medium at 37 °C, in dynamic stirring conditions). Such a comparative study in a relevant environment has not been reported by now.

The cubic scaffolds investigated in the present study exhibited higher initial stiffness and strength than the gyroid scaffolds, reflecting the greater mechanical efficiency of lattice architectures with discrete load-bearing struts. This behavior is consistent with previous reports showing that conventional lattice designs generally provide higher mechanical performance than more porous biomimetic architectures of comparable dimensions [55,56]. The lower initial mechanical performance of the gyroid scaffolds can be attributed to their highly interconnected TPMS architecture and higher porosity, which promote fluid accessibility but reduce the effective load-bearing cross-section. Similar architecture-dependent trends have been reported for TPMS scaffolds in previous studies [57]. However, when considered in the context of FFF-based manufacturing, the increased geometric complexity of TPMS structures also introduces additional constraints. The present results further indicate that the continuous curvature and spatially varying wall thickness of TPMS structures make these architectures more sensitive to printing resolution, filament deposition stability, and local thermal history during fabrication. Consequently, even when designed with equivalent characteristic dimensions, TPMS scaffolds may exhibit higher effective porosity and localized thinning compared to lattice counterparts, which in turn can influence both their initial mechanical response and their degradation behavior.

Importantly, the present study enables a direct comparison between cubic and gyroid architectures manufactured under identical processing conditions, material system, and length scale. This controlled comparison isolates architecture as the primary variable governing performance differences, in contrast to many previous studies where material formulation, printing parameters, or scaffold scale vary simultaneously, complicating direct interpretation of structure–property relationships. It should be noted that the observed structure–property relationships reflect the combined influence of scaffold architecture, porosity, and relative density, as these parameters are inherently coupled in additively manufactured porous structures. Therefore, the present comparison should be interpreted as an evaluation of the overall architectural design effect rather than the isolated contribution of a single geometrical parameter.

In this context, the structural differences reported in the present work should be interpreted not only as geometric variations, but as fundamentally different load-bearing and transport topologies. The cubic architecture concentrates mechanical load transfer through discrete junctions and struts, whereas the gyroid architecture distributes stresses continuously along curved surfaces. This distinction explains the different degradation trajectories observed during immersion, as progressive fluid uptake and polymer softening altered the load-transfer pathways differently in the two architectures. The SEM observations support this interpretation by showing distinct degradation mechanisms for the two architectures. Cubic scaffolds mainly exhibited localized cracking and strut fracture, whereas gyroid scaffolds underwent progressive plasticization, fragmentation, and loss of structural fidelity (Figure 2).

### 4.2. Degradation-Driven Structure–Property Evolution Under Physiologically Relevant Media

#### 4.2.1. Architecture- and Porosity-Controlled Transport and Degradation Kinetics

Beyond its influence on initial mechanical performance, scaffold architecture also controls degradation behavior by modifying fluid transport pathways and polymer exposure to the surrounding medium. In the present study, the cubic and gyroid scaffolds were fabricated using identical material systems and nominal length scales, allowing architecture to be isolated as the primary variable influencing degradation behavior. In the cubic architecture, the lattice of discrete struts and well-defined junctions results in spatially localized fluid penetration and mechanically concentrated load transfer regions. In contrast, the gyroid scaffold is defined by continuous, non-self-intersecting surfaces and a highly interconnected porous network. This latter geometry promotes more homogeneous fluid accessibility and increases effective surface area exposure.

Previous studies have demonstrated that increasing scaffold porosity significantly accelerates the degradation kinetics and mechanical deterioration in PLA systems. Zohoor et al. [58] reported that a 20% increase in porosity leads to measurable increases in mass loss and substantial reductions in elastic modulus under hydrolytic conditions, emphasizing the strong sensitivity of PLA degradation to internal architecture. Although their work was performed under alkaline accelerated degradation conditions, the observed trends highlight the fundamental role of geometry in governing degradation rates. Within this framework, the more open and continuously accessible network of the gyroid scaffold is expected to facilitate faster fluid transport and more uniform exposure to hydrolytic conditions, thereby promoting progressive and distributed material degradation. Conversely, cubic architecture may initially restrict fluid penetration into internal regions, leading to delayed observable mass loss during early immersion stages.

#### 4.2.2. Influence of Physiological Medium Composition and Dynamic Conditions

Beyond geometric factors, degradation behavior is strongly influenced by the composition and dynamics of the surrounding environment. Unlike simplified degradation media such as saline-based simulated body fluids (SBF) or alkaline solutions commonly used in accelerated degradation studies, the present work employs a protein-rich α-MEM-based medium under continuous dynamic agitation at physiological temperature. This distinction is critical, as protein-containing media introduce additional interfacial phenomena that are absent in simplified ionic solutions. In particular, protein adsorption onto polymer surfaces can lead to apparent mass increases that do not directly reflect polymer degradation [59]. Recent studies have demonstrated that PLA scaffolds exposed to FBS-containing culture media undergo significant protein adsorption, resulting in the formation of a deposited protein layer on the scaffold surface [60]. Therefore, in the present study, the mass increase observed for cubic scaffolds may represent the combined contribution of water uptake and adsorption of organic components from the protein-rich medium, although these contributions were not individually quantified. Furthermore, dynamic stirring conditions enhance convective transport, increasing the frequency of scaffold–fluid interactions and potentially accelerating surface-level degradation processes, while the presence of proteins and organic components in the medium may promote adsorption of biological molecules onto the polymer surface, which can influence scaffold mass measurements, surface morphology, and the interaction between the polymer and the surrounding fluid.

Accordingly, the literature findings obtained in SBF or alkaline environments [61] cannot be directly extrapolated to the present system, as they do not account for protein adsorption, complex fluid composition, or dynamic shear conditions. These factors collectively contribute to a more physiologically relevant but mechanistically complex degradation environment.

The differences in weight variation in the two types of scaffolds can be attributed to the concurrent processes of fluid uptake and material degradation occurring during immersion in the protein-rich medium at 37 °C. The scaffold–fluid interaction is inherently dynamic due to continuous rotational motion under stirring conditions, which introduces mechanical agitation and sustained fluid exchange at the scaffold surface. This coupled mechanical–chemical environment promotes degradation phenomena, which appear to dominate in the case of the gyroid architecture.

The pronounced mass loss observed in gyroid scaffolds (Figure 3) suggests a strong dependence of degradation kinetics on scaffold geometry. The gyroid structure, as previously mentioned, facilitates localized stress concentrations and accelerated surface erosion. Post-extraction observations revealed microcracks and detached fragments on the outer layers of these scaffolds, supporting the occurrence of surface-driven material loss. Under prolonged immersion, PLA chains at the surface become plasticized, leading to increased molecular mobility and enhanced susceptibility to detachment under hydrodynamic loading. This leaching process results in the release of polymer fragments into the surrounding medium. In addition, material loss increases the exposed surface area, which may further accelerate hydrolytic degradation processes.

In contrast, the cubic scaffolds exhibit a net mass increase over the duration of the experiment, which is primarily attributed to fluid absorption (Figure 3). This behavior can be explained by the intrinsic microstructural characteristics of fused filament fabrication (FFF) processing, during which layer-by-layer deposition induces the formation of micro voids and interfacial discontinuities within the polymer network. These microstructural features act as pathways for fluid penetration once the scaffolds are immersed in the aqueous environment. Upon immersion, liquid molecules infiltrate these voids and subsequently diffuse into the polymer matrix, driven by concentration gradients. This results in progressive swelling of the struts as water molecules occupy both interstitial voids and amorphous regions of the polymer. The observed mass increase therefore reflects the dominance of fluid uptake over material degradation within the investigated time frame. It should also be acknowledged that, because degradation was performed in a protein-rich cell culture medium, adsorption of serum proteins onto the PLA surface may have contributed to the measured mass increase. Such protein deposition partially mask the early stages of polymer mass loss. This phenomenon may have acted together with moisture uptake to influence the measured mass variation, particularly for the cubic scaffolds, which exhibited negligible degradation over the investigated period. Water absorption is an early-stage phenomenon in hydrolytically degradable polymers and plays a dual role: it contributes to hydrolytic chain scission while simultaneously masking early-stage mass loss through progressive moisture accumulation within the scaffold structure. Therefore, apparent mass variation measured during immersion represents the combined contribution of water uptake, polymer degradation, and possible release of degraded fragments, rather than degradation alone. Previous studies have demonstrated that fluid uptake occurs rapidly in PLA during immersion, with absorbed fluid detectable within the polymer matrix after the first day of exposure under dynamic conditions [36].

It should be noted that the degradation assessment performed in the present study primarily evaluates macroscopic and structural manifestations of PLA degradation, including mass variation, morphological evolution, and mechanical deterioration. Therefore, the exact molecular evolution of PLA hydrolysis, such as the progression of ester bond cleavage and changes in molecular weight, cannot be directly quantified from the applied characterization techniques. Nevertheless, the combined analysis of mass variation, SEM observations, and mechanical properties provides information regarding the dominant degradation phenomena affecting scaffold functionality. During the early stages of immersion, water uptake and polymer plasticization are expected to dominate, leading to increased molecular mobility and reduction in stiffness. With prolonged exposure, hydrolytic degradation and architecture-dependent material loss become increasingly significant, particularly in the highly interconnected gyroid structures, where enhanced fluid accessibility promotes surface deterioration, fragmentation, and loss of mechanical integrity. Future studies incorporating molecular-level characterization, such as monitoring changes in PLA molecular weight and dispersity during degradation, could provide further insight into the relationship between hydrolytic chain scission and the observed architecture-dependent evolution of scaffold properties. Such analyses would complement the present findings by linking polymer-level degradation mechanisms with macroscopic structural and mechanical responses.

#### 4.2.3. Coupling Between Morphological Damage and Mechanical Deterioration

The morphological evolution observed via SEM provides direct evidence of architecture-dependent degradation mechanisms. In the cubic scaffolds, degradation is primarily characterized by localized crack initiation at strut junctions, followed by progressive fracture propagation and eventual structural discontinuity. This behavior is consistent with stress concentration-driven failure mechanisms typical of lattice architectures. In contrast, gyroid scaffolds exhibit a more distributed form of morphological evolution, characterized by progressive smoothing of curved surfaces and gradual loss of TPMS fidelity rather than discrete fracture events. This behavior reflects a continuous structural attenuation driven by the geometry’s interconnected load pathways, whereas cubic lattices demonstrate higher mechanical resistance. These morphological changes are strongly correlated with the observed mechanical behavior (Figure 6). Both types of scaffold exhibit time-dependent reductions in elastic modulus and compressive strength; however, the rate and functional form of degradation differ between architectures. The cubic scaffolds display a more gradual loss of stiffness followed by abrupt structural failure at later stages, whereas the gyroid scaffolds exhibit a more progressive and continuous mechanical decay throughout the immersion period.

These differences highlight the coupling between microstructural evolution and macroscopic mechanical response, where architecture governs not only initial load distribution but also the trajectory of mechanical degradation under hydrolytic and mechanically assisted aging conditions.

Water absorption is evident also in the cubic structure, and it is likely that the gyroid specimens experience even faster fluid uptake during the initial stages of immersion, which may accelerate their degradation response. Notably, the reduction in mechanical performance of gyroid scaffolds becomes more pronounced after approximately one week of immersion in cell culture medium. This behavior can be explained by the simultaneous occurrence of two competing microscale mechanisms [62]. On one hand, micro voids present at the surface of the PLA fibers become progressively filled with liquid, reducing surface discontinuities and temporarily increasing structural continuity, which may contribute to a transient stabilization or increase in stiffness, like in the case of the cubic scaffolds. On the other hand, ongoing moisture diffusion into bulk material induces plasticization, which reduces stiffness by increasing molecular mobility and facilitating deformation under load, as it happens with the gyroid samples that have a larger area exposed to the fluid.

During the first week of immersion, these two processes counterbalance each other, leading to an equilibrium state that is reflected in the stabilization of the elastic modulus of the day 7 gyroid specimens at a value of 69 MPa, identical to that of the dry condition. After this initial period, plasticization becomes the dominant mechanism, resulting in a progressive decrease in both stiffness and compressive strength. Furthermore, micro voids act as stress concentration sites within the scaffold surface, promoting crack initiation and propagation when external loads exceed local intermolecular bonding forces. The appearance of cracks and detached PLA fragments in the gyroid structure is therefore indicative of advanced mechanical degradation under immersion conditions.

The observed mechanical degradation suggests that cubic architecture primarily undergoes moisture-driven and defect-mediated deterioration, where water uptake and stress concentration effects jointly govern the evolution of its mechanical properties during immersion. The presence of manufacturing-induced surface imperfections, such as micro voids and filament-scale defects, provides preferential sites for microcrack initiation and propagation, accelerating localized damage. In the case of cubic scaffolds, the reduction in elastic modulus and compressive strength is associated with rapid fluid infiltration through the highly open structure and the progressive weakening of load-bearing struts at exposed surfaces and nodal junctions. Owing to the discontinuous nature of the cubic lattice, mechanical integrity is strongly dependent on strut connectivity, making the structure particularly sensitive to localized damage accumulation. Absorbed water molecules may contribute to PLA plasticization, increasing molecular mobility and promoting softening and deformation under applied stresses. This combined effect leads to a progressive loss of structural integrity and a time-dependent reduction in stiffness and strength during immersion.

Gyroid architecture exhibits a more distributed and continuous degradation mechanism, governed by fluid infiltration through a fully interconnected triply periodic minimal surface (TPMS) network. The smooth, curvature-driven geometry promotes spatially homogeneous exposure of the polymer matrix to the surrounding medium, enabling rapid and widespread fluid–material interaction rather than localized penetration. As a result, the exponential decrease observed in both elastic modulus and compressive strength reflects a progressive and system-wide structural attenuation rather than failure dominated by discrete strut collapse. In this architecture, load transfer occurs through continuously curved surfaces, allowing mechanical stresses to be redistributed across the network; however, this same continuity also facilitates uniform plasticization of the PLA matrix upon fluid uptake. Water molecules act as plasticizers, increasing polymer chain mobility and reducing intermolecular interactions across the entire TPMS structure. Consequently, the gyroid scaffold undergoes gradual softening and continuous loss of geometric fidelity, manifested as a continuous decay in mechanical properties over immersion time.

Overall, the experimental observations demonstrate that the degradation-induced mechanical evolution results from the combined action of scaffold architecture, fluid transport, and dynamic exposure. The SEM images revealed the progressive development of architecture-specific damage mechanisms, the mass variation measurements quantified the different degradation kinetics of the two scaffold designs, and the compression tests showed the corresponding reduction in stiffness and strength. Together, these results indicate that the cubic scaffolds predominantly experienced moisture-assisted plasticization and localized damage accumulation, whereas the gyroid scaffolds underwent more extensive fluid infiltration, mass loss, and distributed structural deterioration. Consequently, the evolution of the mechanical properties cannot be interpreted solely in terms of polymer degradation but rather as the combined outcome of material chemistry, scaffold topology, fluid transport, and the dynamic physiological-like environment.

### 4.3. A Clinical Perspective

From a clinical standpoint, the successful application of biodegradable bone scaffolds requires a careful balance between mechanical support, biological integration, and degradation kinetics. Bone regeneration is a dynamic process in which scaffold functionality must evolve together with tissue formation. Following injury, early osteogenesis and vascular infiltration occur within the first weeks of healing, whereas complete remodeling and maturation of newly formed trabecular bone may extend over several months or even years depending on defect size, anatomical location, and patient-specific factors. Therefore, an ideal scaffold should provide sufficient mechanical stability during the initial healing phase while gradually transferring load to the regenerating tissue as the biological structure matures.

In this context, scaffold architecture represents a critical design parameter because it simultaneously influences mechanical performance, fluid transport, cellular infiltration, and tissue development. Recent advances in additive manufacturing have enabled the fabrication of highly controlled porous architectures in which parameters such as pore size, geometry, distribution, and interconnectivity can be tailored according to the requirements of specific clinical applications [63]. Such approaches highlight that scaffold optimization cannot be based on a single performance criterion, but rather requires the integration of structural, mechanical, and biological considerations. Furthermore, the development of computationally assisted design strategies provides new opportunities for generating patient-specific scaffolds adapted to defect geometry and functional requirements, reducing empirical approaches and improving reproducibility in scaffold fabrication [64].

Within this framework, gyroid-based TPMS architectures have attracted considerable attention because their continuous and highly interconnected geometry resembles the structural organization of trabecular bone. It should be noted that geometric biomimicry alone does not necessarily provide biological bioactivity. In clinical translation, TPMS architectures are commonly combined with bioactive materials or surface functionalization strategies [38,65] to enhance osteogenic signaling and bone–implant interactions. Therefore, the advantages associated with gyroid architecture should be considered primarily from a structural and transport perspective, while biological performance depends additionally on material composition and biofunctionalization strategies.

In gyroid structures, the absence of discrete strut junctions, combined with a high surface-to-volume ratio and open transport pathways, provides favorable conditions for fluid exchange, cell migration, and vascular development. Experimental and preclinical studies have demonstrated the regenerative potential of gyroid scaffolds. For example, hydroxyapatite gyroid TPMS scaffolds evaluated in a clinically relevant sheep femoral defect model exhibited effective osteointegration, mineralized tissue formation, and overall bone regeneration performance comparable to established bone substitute materials [66]. Similarly, gyroid titanium alloy scaffolds have been shown to promote enhanced angiogenesis and osteogenesis compared with conventional cubic architectures, demonstrating increased vascular formation and new bone deposition within defect areas [67].

Nevertheless, the biological advantages associated with highly biomimetic architecture must be considered together with their mechanical implications. The present study demonstrates that the gyroid architecture provides a more biomimetic and interconnected structure but exhibits reduced initial stiffness and accelerated mechanical deterioration under dynamic immersion conditions. Conversely, conventional cubic architecture maintains superior mechanical competence and structural integrity during degradation, although with a less biomimetic organization. These findings emphasize that scaffold selection should be dictated by the clinical scenario rather than by the assumption that one architecture is universally optimal. For mechanically demanding defects, maintaining structural support during the early stages of healing may represent the primary requirement, whereas in applications where biological integration and tissue infiltration are dominant considerations, highly interconnected biomimetic architectures may provide significant advantages. For example, cubic architecture may be more suitable for applications where early mechanical stability is critical, such as load-bearing regions of large bone defects or reconstruction scenarios involving substantial physiological forces. Conversely, gyroid-based architectures may be advantageous in applications where enhanced biological integration, vascularization, and tissue infiltration are prioritized, such as metaphyseal bone defects or non-critical defects requiring rapid tissue ingrowth. However, the selection of scaffold architecture should ultimately depend on the specific anatomical location, defect size, loading conditions, and expected regeneration timeline.

Future research directions should therefore focus on the development of multifunctional and adaptive scaffolds capable of simultaneously addressing mechanical and biological requirements. Strategies combining patient-specific computational design, graded architectures, bioactive modifications, and predictive modeling may enable the fabrication of scaffolds with tailored degradation profiles and region-specific mechanical properties. Furthermore, long-term in vivo investigations correlating scaffold degradation, mechanical evolution, vascularization, and bone remodeling remain necessary to establish reliable design criteria for clinical translation of next-generation biomimetic scaffolds.

## 5. Conclusions

The present study investigated the degradation-induced evolution of mechanical performance in 3D-printed cubic and gyroid PLA scaffolds during 21 days of immersion under physiologically relevant dynamic conditions. The results showed that scaffold topology influenced mass variation, morphological evolution, and mechanical retention in a protein-rich environment. The main conclusions of the study are summarized as follows:

- The initial mechanical performance was strongly architecture-dependent. The cubic scaffolds exhibited substantially higher mechanical properties than the gyroid structures, with an approximately 210% higher elastic modulus and more than four-fold higher compressive strength in the non-immersed state. This behavior was associated with their lower porosity and more efficient load-transfer pathways.
- The degradation response was strongly architecture-dependent. Cubic scaffolds maintained relatively stable mass throughout immersion, with fluid uptake dominating over material loss, whereas gyroid scaffolds exhibited progressive mass reduction due to enhanced fluid accessibility and architecture-dependent degradation. Moisture uptake contributed to mechanical deterioration in both structures, but its effect was more pronounced in the gyroid architecture due to increased fluid–material interaction.
- SEM observations and mechanical testing revealed different degradation mechanisms. Cubic scaffolds mainly exhibited localized crack initiation and strut fracture, leading to gradual mechanical deterioration, whereas gyroid scaffolds showed progressive structural deterioration, fragmentation, and more pronounced mechanical decay.
- The Residual Property Model successfully predicted the evolution of the cubic scaffolds, where moisture uptake represented the dominant mechanism affecting mechanical performance. However, its applicability was limited for gyroid scaffolds, where mass loss and topology-driven structural deterioration contributed significantly to property degradation.

From a clinical perspective, the findings indicate that scaffold architecture should be selected according to the balance between mechanical stability, degradation behavior, and biological requirements of the intended application.

Future research should therefore focus on the development of multifunctional scaffolds combining biomimetic architectures with improved mechanical resilience, bioactive modifications, predictive modeling approaches, and advanced three-dimensional characterization techniques, such as micro-computed tomography, to quantify the manufactured pore architecture and establish correlations between effective porosity, degradation kinetics, and mechanical performance, ultimately enabling clinically relevant degradation–regeneration profiles as well as cytocompatibility and medium-related biological assessments to complement the current mechanical degradation analysis. Furthermore, future studies may further investigate scaffold degradation under combined chemical and cyclic mechanical loading conditions to better simulate the complex loading environment experienced in vivo.

## Figures and Tables

**Table 1:** Mass of reference cubic and gyroid samples before immersion.

Cubic Scaffolds	Mass (g)	Gyroid Scaffolds	Mass (g)
1	0.489	1	0.222
2	0.485	2	0.202
3	0.487	3	0.211
4	0.488	4	0.207
5	0.489	5	0.204
6	0.500	6	0.212
7	0.510	7	0.203
8	0.497	8	0.217
9	0.503	9	0.207

**Table 2:** Scaffold variation in percentages at different points in time.

Time (Days)	Gyroid Scaffolds	Weight Variation (%)	Cubic Scaffolds	Weight Variation (%)
7	1, 2, 3	−4.09 ± 4.10	1, 2, 3	+0.75 ± 0.06
14	4, 5, 6	−7.06 ± 13.41	4, 5, 6	+0.73 ± 0.07
21	7, 8, 9	−12.8 ± 8.96	7, 8, 9	+1.40 ± 0.13

**Table 3:** Average apparent compression modulus and apparent compressive strength values for the reference scaffolds.

Reference Specimens	Apparent Compression Modulus	Apparent Compressive Strength
Cubic scaffolds	213.7 MPa	20.62 MPa
Gyroid scaffolds	68.9 MPa	4.25 MPa

**Table 4:** Reduced experimental results versus RPM predictions.

Time (Days)	Experimental Results	RPM Prediction	Percent Difference	Experimental Results	RPM Prediction	Percent Difference
7	0.97059	0.9794	0.89927	0.89535	0.92254	2.9478
14	0.98133	0.97977	−0.15835	0.87742	0.92404	5.04526
21	0.95985	0.97032	1.07935	0.83333	0.88523	5.86292

## Data Availability

The datasets generated and/or analyzed during the current study are available from the corresponding author upon reasonable request.

## References

[1] Kozaniti F.K., Deligianni D.D., Georgiou M.D., Portan D.V. (2022). The role of substrate topography and stiffness on MSC cells functions: Key material properties for biomimetic bone tissue engineering. Biomimetics.

[2] Portan D.V. Biomimetic biomaterials: Functioning principles, key properties and perspectives. Proceedings of the Book of Abstracts of the 10th International Conference on Structural Analysis and Advanced Materials (ICSAAM 2023).

[3] Liu H., Liu J., Sun C., Wang Y., Sun Y., Shi X. (2026). Design and fabrication of biomimetic gradient bone tissue engineering scaffolds: Evolution from single-gradient to multi-gradient. Gels.

[4] Pan C., Yang N., Zhang Q., Chen J., Liu Y., Zheng Q. (2026). Dual-biomimetic hydrogel coatings inspired by cell membrane and extracellular matrix: Boosting corrosion resistance and biocompatibility of magnesium alloys. Prog. Org. Coat..

[5] Odabasi R., Krattiger L.A., Kanari M., Jacobs M.A., Moser L.O., Tibbitt M.W., Halin C., Ehrbar M. (2026). Bioengineered lymphatic vessels in synthetic matrices to study breast cancer cell functions. Adv. Healthc. Mater..

[6] Yao K., Xia P., Kong W., Liu N., Lv S., Zhang Y., Yuan X., He J., Ouyang H., He Y. (2026). 3D printing of gradient biomimetic scaffold via electrochemical molecular lock for tissue regeneration. Adv. Mater..

[7] Sun W., Lin Q., Zhao B., Li M., Xiao J., Jin X., Liu J., Wang Y., Zhang R., Li X. (2026). Lymphatic incorporated biomimetic scaffold enhances osteoangio-lymphogenic coupling via HIF-1α-mediated mitochondrial reprogramming for osteoporotic bone repair. Bioact. Mater..

[8] Farshbaf Shakib R., Haghbin Nazarpak M., Solouk A., Bahrami S. (2026). Biomimetic multi-layer scaffolds with aligned polyurethane nanofibers and tailored hyaluronic acid/gelatin hydrogels for sustained methylprednisolone release in potential cardiovascular applications. RSC Adv..

[9] Wang B., Wang L., Yuan T., Zhang Y., Yang Q., Ou H., Zhang B., Yang L., Li S. (2026). Biomimetic microgrooved methacrylated silk fibroin cartilage scaffold for tracheal injury repair. Adv. Funct. Mater..

[10] Das P.J., Sajayan K., Devakate S., Kapat K. (2026). Engineering next-generation vascular grafts with improved endothelialization and long-term patency. Adv. Healthc. Mater..

[11] Zhang Y., Yang Y., Xie R., Wang Z., Lu P. (2026). Structural design of tough porous materials: From natural to biomimetic. Small.

[12] Xia S., Zhao M., Hu L., Ma S., Deng H., Dong X., Sa Y. (2026). Bamboo-based biomass materials with biomimetic structure and osteoconductive capability for bone regeneration. Chem. Eng. J..

[13] Yu M., Chen L., Cai X., Cui S., Li Z., Ding C., Zhang H., Liu H., Li C., Zhang S. (2026). Endogenous metal ion-enriched immunostimulating biomimetic scaffold improves in situ bone regeneration. Adv. Mater..

[14] Liu D., Liu J., Zhao P., Peng Z., Geng Z., Zhang J., Zhang Z., Shen R., Li X., Wang X. (2024). 3D bioprinted tissue-engineered bone with enhanced mechanical strength and bioactivities: Accelerating bone defect repair through sequential immunomodulatory properties. Adv. Healthc. Mater..

[15] Men Y., Wang T., Tang S., Zhang C. (2026). Research on parametric construction and compressive mechanical properties of biomimetic bone trabecular porous scaffold based on Voronoi tessellation. Comput. Methods Biomech. Biomed. Eng..

[16] Zhang J., Chen H., Li X., Zhang A., Liu Y., Wang J., Zhao X., Han Q. (2026). Recent advances in 3D printing biomimetic gradient scaffolds for tendon-bone interface repair. Mater. Today Adv..

[17] Shin H., Jo S., Mikos A.G. (2003). Biomimetic materials for tissue engineering. Biomaterials.

[18] Portan D.V., Deligianni D.D., Kroustalli A.A., Tyllianakis M., Papanicolaou G.C. (2018). Modeling of the interaction between osteoblasts and biocompatible substrates as a function of adhesion strength. J. Biomed. Mater. Res. A.

[19] Papanicolaou G.C., Charitidis C.A., Portan D.V., Perivoliotis D.K., Koklioti M.A. (2014). Investigation of nanomechanical properties of multilayered hybrid nanocomposites. Meccanica.

[20] Portan D.V., Papanicolaou G.C., Jiga G., Caposi M. (2012). A novel experimental method for obtaining multi-layered TiO_2_ nanotubes through electrochemical anodizing. J. Appl. Electrochem..

[21] Portan D.V., Papaefthymiou K., Pirvu C., Papanicolaou G.C., Demetrescu I. (2012). Manufacturing and characterization of TiO_2_ nanotubes on pure titanium surfaces for advanced biomedical applications. UPB Sci. Bull. Ser. B Chem. Mater. Sci..

[22] Zhong J., Wu F., Yang M., Zhao C., Huang C., Chen H., Zhou Z., Lv L., Zhang C., Mao Z. (2026). Biomimetic multifunctional scaffolds for osteochondral regeneration: Bridging material design and functional integration. Small.

[23] Hollister S.J. (2005). Porous scaffold design for tissue engineering. Nat. Mater..

[24] Place E.S., Evans N.D., Stevens M.M. (2009). Complexity in biomaterials for tissue engineering. Nat. Mater..

[25] Haryati E., Lolo J.A., Dinatha I.K.H., Juliadmi D., Kusumaatmaja A., Partini J., Ana I.D., Syahruddin M.H., Wihadmadyatami H., Nuryanti A. (2026). Biogenic hydroxyapatite derived from Polymesoda erosa shells tunes structure–property–performance in 3D-printed PLA/PCL scaffolds for bone tissue engineering. Next Mater..

[26] Büyük N.İ., Hacıhasanoğlu E., Çiçek Ç., Köse G.T. (2026). Advanced 3D-printed PLA scaffolds functionalized with dual small molecules for enhanced bone regeneration: In vitro and in vivo studies. Biomater. Sci..

[27] Bose D., Nag P., Dhal M.K., Katiyar V. (2026). PLA-based composite implants for mandibular fracture: Towards bioresorbable solutions. J. Mech. Behav. Biomed. Mater..

[28] Xu Y., Peng W., Poli V., Lavagnolo M.C., Kalambura S., He P.-J., Lü F., Zhang H. (2026). A review of the degradation mechanisms of biodegradable PLA/PBAT films: Biological, chemical, and biochemical perspectives. Energy Environ. Sustain..

[29] Saidin S., Zubairi S.I., Ambreen J., Lazim N.A.M., Sadia M., Lim J.T.W., Ulum M.F., Elliyanti A., Sefat F. (2026). Biodegradable synthetic polymers for biomedical and tissue engineering applications: Tailoring degradation kinetics with tissue regeneration timeline. BioMed. Eng. OnLine.

[30] Aydin M.S., Yamada S., Mohamed-Ahmed S., Rashad A., Mustafa K. (2026). Influence of scaffold porosity on the degradation of 3D-printed biodegradable polymers: Coupled bioreactor, modeling, and in vivo validation. Mater. Today Adv..

[31] Chen X., Chen G., Wang G., Zhu P., Gao C. (2020). Recent progress on 3D-printed polylactic acid and its applications in bone repair. Adv. Eng. Mater..

[32] Baptista R., Guedes M., Pereira M.F.C., Maurício A., Carrelo H., Cidade T. (2020). On the effect of design and fabrication parameters on mechanical performance of 3D printed PLA scaffolds. Bioprinting.

[33] Bakhtiari H., Nouri A., Aamir M., Najafi M., Tolouei-Rad M. (2025). Impact of biodegradation on the mechanical and fatigue properties of 3D-printed PLA bone scaffolds. J. Mech. Behav. Biomed. Mater..

[34] Pazhamannil R.V., Alkhedher M. (2026). Structure-property relationships in 3D printed bone scaffolds: Role of TPMS architecture and porosity in PLA and nanohydroxyapatite reinforced composites. J. Thermoplast. Compos. Mater..

[35] Kachalina P.M., Kovaleva P.A., Cheremnykh A.I., Statnik E.S., Senatov F.S., Anisimova N.Y. (2026). The effect of the biodegradation process on the structure and mechanical properties of 3D-printed composite materials based on PLA. J. Polym. Res..

[36] Portan D., Angelopoulou A., Koliadima A., Kontaxis L., Michanetzis G., Kouzoudis D., Michalopoulos E., Papanicolaou G. (2025). Biodegradation, mechanical and biocompatibility evaluation of compact and porous polylactic acid after long-term dynamic fluid immersion. J. Chem. Technol. Biotechnol..

[37] Portan D.V., Koliadima A., Kapolos J., Azamfirei L. (2025). Biomimetic design and assessment via microenvironmental testing: From food packaging biomaterials to implantable medical devices. Biomimetics.

[38] Tourbier C., Basoli V., Maintz M., Bella E.D., Stoddart M.J., Thieringer F.M. (2026). Biomimetic 3D-printed gyroid scaffolds with versatile bioactive coatings for complex craniomaxillofacial bone regeneration. Biomed. Mater..

[39] Johnson P., Wayland H. (1967). Regulation of blood flow in single capillaries. Am. J. Physiol..

[40] Papanicolaou G.C., Zaoutsos S.P., Anastasiou D.E. (2022). Manufacturing technology and dynamic mechanical investigation of moisture-resistant *Luffa cylindrica*-reinforced epoxy composites. J. Appl. Polym. Sci..

[41] Papanicolaou G.C., Kontaxis L.C., Koutsomitopoulou A.F., Zaoutsos S.P. (2015). Stress relaxation behavior of starch powder–epoxy resin composites. J. Appl. Polym. Sci..

[42] Kollia A., Kontaxis L.C., Papanicolaou G.C., Zaoutsos S.P. (2020). Effect of thermal shock cycling on the quasi-static and dynamic flexural properties of flax fabric–epoxy matrix laminates. J. Appl. Polym. Sci..

[43] Wong K.J., Johar M., Koloor S.S.R., Petrů M., Tamin M.N. (2020). Moisture absorption effects on mode II delamination of carbon/epoxy composites. Polymers.

[44] Banjo A.D., Agrawal V., Auad M.L., Celestine A.-D.N. (2022). Moisture-induced changes in the mechanical behavior of 3D printed polymers. Compos. Part C Open Access.

[45] Saracoglu G. (2023). The modified residual property model for fracture mechanics. Mechanika.

[46] Wong K.J., Israr H.A., Tamin M.N. (2026). Effect of water absorption on mode II delamination of adhesively bonded carbon/epoxy composites. J. Fail. Anal. Prev..

[47] Karimipour-Fard P., Behravesh A.H., Jones-Taggart H., Pop-Iliev R., Rizvi G. (2020). Effects of design, porosity and biodegradation on mechanical and morphological properties of additive-manufactured triply periodic minimal surface scaffolds. J. Mech. Behav. Biomed. Mater..

[48] Verma S., Singh G., Choudhary A., Hussain S., Chanda A. (2026). Mechanical properties of whole-body human bones: A review. Mater. Res. Express.

[49] Rice J.C., Cowin S.C., Bowman J.A. (1988). On the dependence of the elasticity and strength of cancellous bone on apparent density. J. Biomech..

[50] Elenskaya N., Tashkinov M., Silberschmidt V.V. (2025). TPMS-based scaffolds: Adaptation of morphological properties and mechanical response to reference tissue. Int. J. Solids Struct..

[51] Fyhrie D., Schaffler M. (1994). Failure mechanisms in human vertebral cancellous bone. Bone.

[52] Serra T., Planell J.A., Navarro M. (2013). High-resolution PLA-based composite scaffolds via 3-D printing technology. Acta Biomater..

[53] Huang Y., Mao Y., Li H., Wang E., Mai H., Zhang W., Wen J., You H., Long Y., Guo W. (2025). 3D-printed thermally activated shape memory PLA/TBC composite scaffold with body-compatible temperature for minimally invasive bone repair. ACS Appl. Polym. Mater..

[54] Kwon S., Hwang D. (2025). Understanding and resolving 3D printing challenges: A systematic literature review. Processes.

[55] Rahman A., Tanvir K.F., Jui H.K., Ahmed M.T., Nipu S.M.A., Hussain S.B. (2025). Design and analysis of 3D-printed PLA scaffolds: Enhancing mechanical properties. Results Eng..

[56] Hulme J., Sakhaei A.H., Shafiee M. (2024). Mechanical analysis and additive manufacturing of 3D-printed lattice materials for bone scaffolds. Mater. Today Proc..

[57] Verisqa F., Park J.-H., Mandakhbayar N., Cha J.-R., Nguyen L., Kim H.-W., Knowles J.C. (2024). In vivo osteogenic and angiogenic properties of a 3D-printed isosorbide-based gyroid scaffold manufactured via digital light processing. Biomedicines.

[58] Zohoor S., Abolfathi N., Solati-Hashjin M. (2023). Accelerated degradation mechanism and mechanical behavior of 3D-printed PLA scaffolds for bone regeneration. Iran. Polym. J..

[59] Tanaka M., Mochizuki A., Shiroya T., Motomura T., Shimura K., Onishi M., Okahata Y. (2002). Study on kinetics of early stage protein adsorption on poly(2-methoxyethylacrylate) (PMEA) surface. Colloids Surf. A Physicochem. Eng. Asp..

[60] Atay I., Yilgör E., Sürme S., Kavakli I.H., Yilgör I. (2024). Critical role of the composition of the cell culture medium on cell attachment and viability on PLA biocomposite scaffolds under in vitro assay conditions. Polymer.

[61] Zhao W., Lemaître J., Bowen P. (2017). A comparative study of simulated body fluids in the presence of proteins. Acta Biomater..

[62] Theocaris P., Papanicolaou G., Kontou E. (1983). Interrelation between moisture absorption, mechanical behavior, and extent of the boundary interphase in particulate composites. J. Appl. Polym. Sci..

[63] Picado-Tejero D., Mendoza-Cerezo L., Rodríguez-Rego J.M., Carrasco-Amador J.P., Marcos-Romero A.C. (2025). Recent Advances in 3D Bioprinting of Porous Scaffolds for Tissue Engineering: A Narrative and Critical Review. J. Funct. Biomater..

[64] Logeshwaran A., Elsen R., Nayak S. (2024). Artificial Intelligence-Based 3D Printing Strategies for Bone Scaffold Fabrication and Its Application in Preclinical and Clinical Investigations. ACS Biomater. Sci. Eng..

[65] Vijaya Kumari P.K., Carpenter J., Cleon B., Panebianco C.J., Boerckel J.D., Dean D., Vijayan V.M. (2026). Plasma-enabled multiscale coupling of architecture and biointerfaces drives osteogenesis in 3D-printed gyroid scaffolds. bioRxiv.

[66] Bouakaz I., Drouet C., Grossin D., Cobraiville E., Nolens G. (2023). Hydroxyapatite 3D-Printed Scaffolds with Gyroid-Triply Periodic Minimal Surface (TPMS) Porous Structure: Fabrication and an In Vivo Pilot Study in Sheep. Acta Biomater..

[67] Wang L., Wang Y., Liu R., Liang Y., Liu Y., Xu M., Yu J., Su Y., Han Z., Wang X. (2026). An experimental study on 3D-printed gyroid-shaped TC4 porous scaffolds guiding angiogenesis and osteogenesis in bone defect areas. ACS Biomater. Sci. Eng..

